# The development of a decision aid for shared decision making in the Dutch implantable cardioverter defibrillator patient population: A novel approach to patient education

**DOI:** 10.3389/fcvm.2022.946404

**Published:** 2022-10-13

**Authors:** Dilek Yilmaz, Anastasia D. Egorova, Martin J. Schalij, Han A. M. Spierenburg, Robert A. M. Verbunt, Lieselot van Erven

**Affiliations:** ^1^Department of Cardiology, Leiden University Medical Center, Leiden, Netherlands; ^2^Department of Cardiology, Sint Maarten Medical Center, Cay Hill, Sint Maarten; ^3^Department of Cardiology, Maxima Medical Center, Eindhoven, Netherlands

**Keywords:** cardiac geriatrics, implantable cardioverter-defibrillator, shared decision making, decision aid (DA), decision making

## Abstract

**Background:**

Counseling of Implantable Cardioverter-defibrillator (ICD) patients with regard to individual risks and benefits is challenging. An evidence-based decision aid tailored to the needs of Dutch ICD patients is not yet available. The objective of this pilot project was to structurally evaluate the current clinical practice in The Netherlands and the ICD patient experience, in order to develop an online decision aid to facilitate shared decision making in ICD procedures.

**Methods:**

Between June 2016 and December 2017, a Dutch web-based decision aid was developed according to the Patient Decision Aid Standards (IPDAS) using the RAND-UCLA/multi-stepped Delphi model. Development process consisted of 5 stages in which the Dutch clinical practice was reviewed (stage 1), patients’ needs and their history of decision making was structurally assessed (stages 2A and B) and a modified Delphi consensus process was performed with an expert panel consisting of representatives from different medical fields (stage 3). Results from stages 1–3 were used to design and structure the content of an online-based decision aid (stage 4) which was finally evaluated in a usability testing by patients in stage 5.

**Results and conclusion:**

This study describes the evidence-based approach to the development of the Dutch ICD decision aid. In our population, levels of shared decision-making experience were low. The ICD decision aid was structurally developed for the Dutch ICD patient population. Our upcoming multicenter stepped wedge clustered randomized trial will further evaluate the ICD decision aid in clinical practice.

## Introduction

A large body of evidence has shown that Implantable Cardioverter-defibrillators (ICD) play an important role in primary and secondary prevention of sudden cardiac death. For secondary prevention, ICD benefit is more clear (1, 2). Nevertheless, the majority (50–90%) of the ICD patient population receives an ICD for primary prevention (3). Benefit in terms of appropriate tachytherapy varies widely within the latter population: from 50% at 3 years follow-up to only 2.4% in a recent meta-analysis for non-ischemic cardiomyopathy patients (4). Despite the increasing number of trials and scientific literature, it remains challenging for individual patients to perceive the impact of an ICD (5) and for medical professionals to appreciate patient’s values and to translate scientific data into individually applicable advantages (6). In addition to potential periprocedural and later complications, ICDs also impose psychological and social consequences on patients and their family (7, 8). This makes patient counseling challenging. The most recent European guideline (2021) on cardiac pacing and cardiac resynchronization therapy stipulates the importance of patient-centered counseling and shared decision making with regard to device implantations (9). Moreover, the American Medicaid insurance policy has mandated shared decision making in patients undergoing cardiac device implantations with the help of evidence-based decision tools (10). An evidence-based decision aid tailored to the needs of Dutch ICD patients is not yet available.

The objective of this pilot project was to structurally evaluate current clinical practice in Netherlands and ICD patient experience, in order to develop an online decision aid that may improve the levels of shared decision making in ICD implantations and pulse generator exchanges and to decrease decisional conflict.

## Materials and methods

**FIGURE 1:**
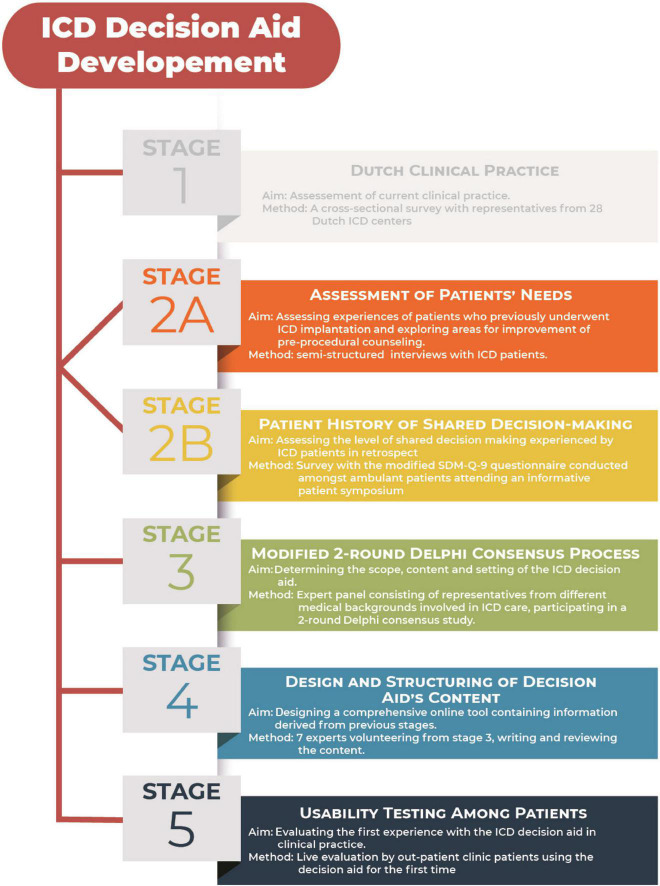
Overview of stages in developing the ICD decision aid.

Between June 2016 and December 2017, a Dutch web-based decision aid was developed according to the Patient Decision Aid Standards (IPDAS) (11) using the RAND-UCLA/multi-stepped Delphi model (12, 13). Development process consisted of 5 stages, illustrated in Figure 1.

*Stage 1: Interview-based evaluation of the Dutch Clinical Practice on Implantable Cardioverter-defibrillators.*

All centers in Netherlands qualified to implant ICDs were contacted (*n* = 28). Representative cardiologists of Dutch ICD implanting centers were interviewed. The results of this study have been recently published (14).

*Stage 2A: Assessment of patients’ needs.*

Ten (10) patients [median age 66 (IQR 52–77) years, 30% female, 50% ICD for primary prevention, 10% previously declined a device, 20% CRT-D and 20% ICD] were interviewed between March and April 2017. Patients were selected at the cardiology outpatient clinic of the Leiden University Medical Center and represented the following categories: patients with an ICD for primary and secondary prevention, patients with a Cardiac Resynchronization Therapy-defibrillator (CRT-D) device and patients who previously declined an ICD implantation. To avoid the potential impact of a medical environment on the in-depth interviews, patients were interviewed at a neutral office outside of the hospital. Semi-structured interviews with questions on their decision making process were performed, inquiring about their reasons for choosing or refraining from an ICD, pre-operative counseling by caregivers and current experiences and needs as an ICD patient. Questions on the survey were designed based on clinical experience and outcome of interest. Desired outcome parameters were predefined. Responses were recorded on audiotape with permission from the participants and transcribed as text. Answers were analyzed by the primary investigator and matched and scored accordingly to the predefined outcome parameter. Participants were invited to propose topics and items which they considered to be valuable for peers to be included in a decision aid.

*Stage 2B: Patient history of shared decision-making.*

A cross-sectional assessment of shared decision-making experience levels was performed in ICD patients attending a biannual ICD patient conference in the Leiden area. All the attending patients (*n* = 245) received questionnaires comprising of questions based on the Dutch SDM-Q-9 (15) (Table 1, questions 5–13). In addition, questions regarding patient demographics, together with two statements of interest for patients who previously had undergone a pulse-generator exchange at the time of battery depletion, were added. Patients indicated their level of agreement on a 5-point Likert Scale. The outcomes were analyzed according to the SDM-Q-9 user manual (15). Questionnaires missing answers to more than 2 questions were excluded from the analysis. In the case of 1 or 2 missing values, these were corrected by imputation: the imputed score was the mean score of the present variables (15). We evaluated the additional questions (questions 14–16) as a percentage by grouping the agreement and disagreement answers.

**TABLE 1:** Modified 9-item Shared Decision-Making Questionnaire (SDM-Q-9) with additional questions for regional ICD patient conference.

Question	Answer options
1. I have	*An ICD – a CRT-D – no device*
2. Age	… *years*
3. Gender	*Female - male*
4. I received my device in the year	….
5. Cardiologist made clear I had a choice.	*Strongly disagree – disagree – neutral - agree-strongly agree*
6. Cardiologist wanted to know how much I wanted to be involved in the decision-making.	*Strongly disagree – disagree – neutral - agree-strongly agree*
7. Cardiologist told me there were other options than an ICD.	*Strongly disagree – disagree – neutral - agree-strongly agree*
8. Cardiologist explained pros and cons.	*Strongly disagree – disagree – neutral - agree-strongly agree*
9. Cardiologist helped me understand all the information.	*Strongly disagree – disagree – neutral - agree-strongly agree*
10. Cardiologist asked me if I preferred an ICD.	*Strongly disagree – disagree – neutral - agree-strongly agree*
11. Cardiologist and I thoroughly reconsidered an ICD.	*Strongly disagree – disagree – neutral - agree-strongly agree*
12. Cardiologist and I chose an ICD together.	*Strongly disagree – disagree – neutral - agree-strongly agree*
13. I felt as if I could choose between an ICD or none.	*Strongly disagree – disagree – neutral - agree-strongly agree*
14. My device has been replaced due to battery depletion.	*Yes – no, never*
15. I could choose not to replace my ICD at the time of battery depletion.	*Strongly disagree – disagree – neutral - agree-strongly agree*
16. An ICD is a life-long commitment/obligation for me.	*Strongly disagree – disagree – neutral - agree-strongly agree*

ICD, Implantable Cardioverter-defibrillator; CRT-D, Cardiac Resynchronization Therapy-defibrillator. Original statements in Dutch.

*Stage 3: Modified 2-round Delphi Consensus Process.*

For determining the content and setting of the decision aid, a modification of the RAND Corporation/University of California, Los Angeles consensus methodology on appropriateness ratings was used as described below (16). A total of 19 experts from different medical centers over the country were to participate in the expert panel for determining the setting and content of the decision aid. The panel consisted of 7 cardiologists, 1 ICD nurse, 2 general practitioners/family medicine doctors, 1 dedicated MD PhD-fellow focusing on ICD patient care, 3 specialists in elderly care medicine/geriatric specialist, 2 internal medicine physicians specialized in elderly medicine, 1 lawyer specialized in medical ethics, 1 psychologist, the chairman of the ICD patient federation and 1 expert on decision aid development. Statements for the experts to evaluate were formulated to determine the content and setting of the decision aid based on information from literature, guidelines and findings from the previous stages (1, 2, 4, 17–24).

Round 1: Participants received an online questionnaire with 84 items divided into 5 categories (1-target group and setting, 2-content, 3-to be included patient preferences, 4-screening and tools, and 5-format of the decision aid) (Supplementary Appendix 2). Nineteen (19) items consisted of yes or no questions and 64 items were statements for which the experts indicated their level of agreement on a 10-point Likert scale (12, 13). Consensus outcomes were classified as median scores. A median score > 7 was considered as positive consensus and the statement was accepted. A median score < 5 was resulted in the rejection of the statement. Scores between 5 and 7 were discussed in the second round to seek consensus. Participants also had the opportunity to add on items they felt were missing from the questionnaire, which could be discussed in the second round.

Round 2: All participants from round 1 were invited for a face-to-face meeting. Statements from the previous questionnaire on which no consensus was yet reached and items added on by individual experts, were put up for discussion one by one. At the end of each discussion, consensus on agreeing or rejecting the statement was reached by popular vote with a 2/3 majority.

*Stage 4: Design and Structuring of decision aid’s content.*

Members of the working group were recruited from the previous expert panel (described in stage 3) in order to form a dedicated team for the materialization of the actual decision aid. Members of the working group consisted of three cardiologists from different hospitals, one decision aid development expert, 1 general practitioner/family medicine physician, one Internist-geriatrician and one dedicated MD PhD-fellow focusing on ICD patient care.

The working group formulated the factual content of the decision aid based on the findings and recommendations from previous stages. Engineers and designers from ZorgKeuzeLab (Delft, Netherlands) designed a functioning web-based tool encasing the information provided by the working group.

*Stage 5: Usability testing of the prototype among patients.*

The four patients from the outpatient clinic with an ICD device were randomly selected and invited to undergo in-depth interviews while testing and analyzing the usability of the prototype of the ICD decision aid. These patients were not involved in the previous stages of the study. Patients were invited for participation by the device technician during their regular semi-annual check-ups. Patients were encouraged to provide live commentary on their experience as they navigated through the decision aid. Patients received open questions addressing whether the decision aid was easy to navigate though, whether they understood the images and animations, whether explanations were clear and easy to read and if they had suggestions for improvement.

### Statistics

Categorical variables were presented as numbers and percentages. Based on their distributions, continuous variables are presented as mean ± standard deviation (SD) or median with interquartile range (IQR) [25th to 75th percentile].

## Results

*Stage 1: Interview-based evaluation of the Dutch Clinical Practice on Implantable Cardioverter-defibrillators.*

Results have recently been published (14).

*Stage 2A: Assessment of patients’ needs.*

The patients’ response rate was 100% (*n* = 10). The mean age was 62 ± 12 years, 90% male, 90% underwent an ICD implantation (70% for primary prevention) and the median time from first ICD implantation to interview was 7.5 [7–16] years. One patient (10%) with an indication for ICD implantation had declined this. Three (30%) patients experienced appropriate shock therapy and two (20%) had received one or more pulse-generator exchanges for battery depletion. Patients reported shocks as unpleasant and painful, however, they were also well accepted. One patient (10%) had experienced inappropriate shock therapy. Patients frequently reported that they experienced not to have had a choice or to have trusted their doctor’s judgment and (strong) recommendations (50%). In addition, all three patients with an ICD for secondary prevention referred to their choice as “choosing between life or death,” whereas primary prevention patients mostly deemed their ICD as an extra insurance (4 out of 7, 57%). One patient (with an ICD for primary prevention) reported to regret the decision, due to limitations in (life-)insurance and traveling opportunities. Furthermore, patients reported implications for their driver’s license as an important downside to having an ICD (60%) (Table 2A).

**TABLE 2A:** Stage 2A: Assessment of patients’ needs.

	*N* = 10
*Male (%)*	90
*Age (mean, SD)*	62 ± 12
*Time from first ICD implantation (median, IQR)*	7.5 [7–16]
*ICD indication for primary prevention (%)*	70
Declined an ICD *(%)*	10
Underwent ≥ 1 pulse generator replacement for battery depletion *(%)*	20
Received appropriate ICD (shock) therapy *(%)*	30
Received inappropriate ICD shock therapy *(%)*	10
Perceived not to have a choice regarding ICD implantation *(%)*	50
“The ICD is an extra insurance” *(%)*	30
Regretting the ICD implantation *(%)*	10
Impaired by driver license restrictions *(%)*	60

ICD, Implantable Cardioverter-defibrillator; N, number of total patients that filled in the specific question(s); SD, Standard Deviation. Original statements in Dutch.

*Stage 2B: Patient history of shared decision-making.*

A total of 233 patients completed the modified SDM-Q-9 questionnaire (95% response rate). The mean age was 69 ± 10 years, 75% male, the median time from first ICD implantation to interview was 5 (IQR 2–10) years and 56% had a CRT-D. Eighty-six respondents (40%) had previously undergone at least once a pulse-generator exchange due to battery depletion. Scores from the modified SDM-Q-9 questionnaires on the level of decision-making could be calculated for 133 respondents (57%). The remaining questionnaires were excluded from analysis due to missing data in accordance with the SDM-Q-9 manual (25). Patients reported to be satisfied with the pre-operative information, however, on a scale of 0 (no shared decision experienced) to 100 (strong shared decision experienced) levels of shared decision were marked at a mean ranked score of 42 (IQR 15.5–78). Furthermore, most of the patients perceived the ICD to be a “lifelong commitment” (69%). Remarkably, 21 (10%) of the respondents wrote an extra note stating: “I did not have a choice” (Table 2B).

**TABLE 2B:** Stage 2B: Patient history of shared decision making.

		N
*Male (%)*	75	233
*Age (mean, SD)*	69 ± 10 years	233
Time from first ICD implantation (median, IQR)	5 (2–10) years	233
*SDM Score (mean rank, IQR)*	42 (15.5–78)	133
“I could choose not to replace my ICD at the time of battery depletion” (% that disagreed)	50	86
“An ICD is a life-long commitment/obligation for me” (% that agreed)	69	86

SDM score: mean rank calculated score from modified SDM-Q-9 questions reflecting the experienced level of shared decision making (SDM) on a scale from 0 to 100. ICD, Implantable Cardioverter-defibrillator; N, number of total patients that filled in the specific question(s). Original statements in Dutch.

*Stage 3: Modified 2-round Delphi Consensus Process.*

*Round 1*

**FIGURE 2:**
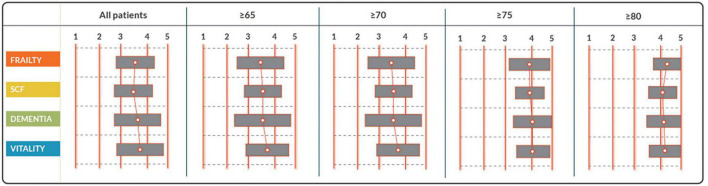
Response to statements on which patients should be screened on what aspects, by tools integrated into the decision aid. Statements are rated on a scale from 1 to 5. Consensus on acceptance is reached with a median score of ≥ 3.5. SCF, social cognitive functioning.

The panel of experts consisted of 7 cardiologists, 1 ICD nurse, 2 general practitioners/family medicine doctors, 1 dedicated MD PhD-fellow focusing on ICD patient care, 3 specialists in elderly care medicine/geriatrics, 2 internal medicine physicians specialized in elderly medicine, 1 lawyer specialized in medical ethics, 1 psychologist, the chairman of the ICD patient federation and 1 expert on decision aid development. Of these experts, 6 were female (32%). The median age was 55 (IQR 43.5–59.5) years and the median clinical experience was 27 (IQR 15.5–30) years. Response rate of the experts on the panel was 100% (*n* = 19). The experts reached a consensus on 56 (86%) statements in the first round. Experts decided that the ICD decision aid was not limited to one category of ICD patients, but should be made available to all patients receiving a first ICD device (*de novo* implants), or who were up for pulse-generator replacement due to battery depletion. In addition, it was agreed that the decision aid would include a tool enabling patients to review their personal preferences. Questions and the corresponding results are found in Tables 3A–E and Figure 2.

**TABLE 3A:** Statements from Delphi round 1 on who should be the target group of the decision aid should be.

Statements	Yes (%)
**The decision aid should be given to …**	
… *all patients receiving an ICD.*	*81*
… *all patients receiving an ICD for the first time.*	*55*
… *all patients who will undergo a pulse-generator replacement due to battery depletion.*	*55*
… *only patients receiving an ICD for primary prevention of sudden cardiac death.*	*36*
… *patients concerning secondary prevention of sudden cardiac death.*	*18*
… *all patients with many comorbidities.*	*55*
… *patients of high age.*	*55*
**The decision aid should be handed out by/made available by…**	
… *the cardiologist*	*91*
… *the general practitioner/family doctor*	*18*
… *the ICD-nurse*	*72*
… *the ICD-technician*	*9*
… *the patient union*	*36*
The decision aid should be handed out *per postal mail*, *before* the consultation with the cardiologist on ICD therapy	9
The decision aid should be handed out *after* consultation with the cardiologist on ICD therapy	91

ICD, implantable cardioverter-defibrillator. Original statements in Dutch. *N* = 19.

**TABLE 3B:** Statements from Delphi round 1 on the content of the decision aid.

Statements	Median	Consensus
General explanation about what an ICD does should be included in the decision aid	9.7	Accepted
Discussion on therapeutically benefits of an ICD with primary prevention patients should be separate from secondary prevention patients	7.6	Accepted
The choice for a subcutaneous ICD should be included in the content	6.2	Deferred
Explanation of Cardiac Resynchronization Therapy (CRT) should be included in the content	5.7	Rejected
The added value of an ICD with patients at an older age should be discussed nuanced	9.8	Accepted
The added value of an ICD with patients with unclear life expectancy should be discussed	9.6	Accepted
The most common complications of a procedure should be discussed	9.6	Accepted
Complications of a prolonged hospitalization, such as pneumonia and decubitus in case of immobilization, should be discussed	4.5	Rejected
Risk on advisory leads and recall products should be included in the explanation by default	5.6	Rejected
The role of the ICD at the end of life should be discussed with all patients	8.2	Accepted
The possibility to deactivate tachytherapy at the end of life should be discussed with all patients	7.3	Accepted
**All** patients should know that an ICD should not be a lifelong commitment	9.3	Accepted
**All** patients should know that an ICD, if not desired, can be turned off	9.8	Accepted
The role of the ICD at the end of life should be discussed with patients of **old** age	9.3	Accepted
The possibility to deactivate tachytherapy at the end of life should be discussed with patients of **old** age	8.9	Accepted
Patients of **old** age should know that an ICD does not have to be a lifelong commitment	9.4	Accepted
Patients of **old** age should know that an ICD, if not desired, can be turned off	9.4	Accepted
The technical aspects of how an ICD works should be included in the counseling material	6.7	Deferred
The benefits of tachytherapy should be explained	7.5	Accepted
It should be **explained** that ICD therapy protects against sudden cardiac death and not against sudden death in general (because of other causes of death)	9.4	Accepted
It should be **stressed** that ICD therapy protects against sudden cardiac death and not against sudden death in general (because of other causes of death	8.5	Accepted
The psychological impact of tachytherapy (more depression, traumatic) should be included in the general content	6.9	Deferred
The chance of inappropriate therapy should be included in the content	8.7	Accepted
How you should resuscitate a patient with ICD should be included to the content	5.2	Rejected
Telemonitoring should be explained	6.7	Deferred
The function of various healthcare specialists, cardiologists, EP-cardiologist, ICD nurses and ICD technicians, should be explained in the content	6.4	Deferred

Statements are rated on a scale from 1 to 10. Consensus on acceptance is reached with a median score of ≥ 7. ICD, implantable cardioverter-defibrillator; EP, electrophysiologist; CRT, cardiac resynchronization therapy. Original statements in Dutch.

**TABLE 3C:** Statements in Delphi round 1 on items to be included in rating scales for patients.

Statements	Median	Consensus
In the decision aid, patients should be able to select on a rating scale…		
… how much they tend to an ICD or not.	3.9	Accepted
… how much they value the advice of their health care provider.	3.9	Accepted
… how much they value the opinion of close ones/relatives.	3.7	Accepted
… how much anxiety they feel for receiving appropriate therapy.	3.6	Accepted
… how much anxiety they feel for receiving inappropriate therapy.	3.7	Accepted
… how self-sustainable they will feel when shock therapy is being felt.	3.6	Accepted
… how self-sustainable they expect to be in showing up on all follow-up appointments.	3.6	Accepted
… how willing they will be to undergo re-inventions for battery replacements.	2.9	Accepted
… how affected they will be by the consequences for their driver’s license after implantation.	4.0	Accepted
… how affected they will be by the consequences for their driver’s license after receiving shock therapy.	3.9	Accepted
… how willing they are to comply with the necessity for at least semi-annual ICD check-ups	3.7	Accepted
… how much anxiety they feel for potential complications	3.5	Accepted
… their value for philosophical elements, such as the role of ICD at the end of life.	3.8	Accepted
… their value for the psychological aspects of shock therapy, such as the probability of depression and decrease in quality of life	3.9	Accepted
… their preference for life extension, such as the role of ICD in the mortal process	4.0	Accepted
… their value for the cosmetic aspects of an ICD, such as the scar and visibility of the contour of the pulse-generator	3.3	Rejected
… their preference for life extension above the quality of life (for instance: understanding that preventing sudden cardiac death can lead to a long hospitalization with heart failure)	4.0	Accepted
…their preference for a non-sudden cardiac death with a potential prolonged death bed.	3.9	Accepted

Statements are rated on a scale from 1 to 5. Consensus on acceptance is reached with a median score of ≥ 3.5. ICD, implantable cardioverter-defibrillator. Original statements in Dutch.

**TABLE 3D:** Statements on which patients should be screened on what aspects, by tools integrated into the decision aid.

Statement	Median	Consensus
With a tool incorporated into the decision aid, ….		
… all patients should be screened on frailty.	3.5	Accepted
… all patients should be screened on social-cognitive functions.	3.4	Rejected
… all patients should be screened on dementia.	3.6	Accepted
… all patients should be screened on vitality.	3.7	Accepted
… patients older than 65 years should be screened on frailty.	3.6	Accepted
… patients older than 65 years should be screened on social-cognitive functioning.	3.7	Accepted
… patients older than 65 years should be screened on dementia.	3.7	Accepted
… patients older than 65 years should be screened on vitality.	3.9	Accepted
… patients older than 70 years should be screened on frailty.	3.6	Accepted
… patients older than 70 years should be screened on social-cognitive functioning.	3.7	Accepted
… patients older than 70 years should be screened on dementia.	3.8	Accepted
… patients older than 70 years should be screened on vitality.	3.9	Accepted
… patients older than 75 years should be screened on frailty.	4.0	Accepted
… patients older than 75 years should be screened on social-cognitive functioning.	4.1	Accepted
… patients older than 75 years should be screened on dementia.	4.2	Accepted
… patients older than 75 years should be screened on vitality.	4.2	Accepted
… patients older than 80 years should be screened on frailty.	4.5	Accepted
… patients older than 80 years should be screened on social-cognitive functioning.	4.3	Accepted
… patients older than 80 years should be screened on dementia.	4.5	Accepted
… patients older than 80 years should be screened on vitality.	4.5	Accepted

Statements are rated on a scale from 1 to 5. Consensus on acceptance is reached with a median score of ≥ 3.5. Original statements in Dutch.

**TABLE 3E:** Statements in Delphi round 1 on how the decision aid should be made available.

Statement	Yes (%)
The decision aid should be available in a paper version	100
The decision aid should be available as a downloadable app	56
Only web-access to the decision aid will be sufficient	9
An interactive decision aid, including videos of patient experiences, is preferable	72
Videos with experiences of other patients do not belong in a decision aid	9

*N* = 19. Original statements in Dutch.

*Round 2*

With the exception of 1 expert, all experts from round 1 were available for participation in round 2. The panelists reviewed all statements and results from round 1 and proceeded in discussions on only the statements on which no consensus had yet been reached. In all cases, this resulted in the unanimous rejection of all items that were up for discussion. Rejected statements resulted in the exclusion of content on subcutaneous ICDs, information on resuscitation with an ICD and the risk of anxiety after receiving shock therapy from the ICD.

*Stage 4: Design and structure of Decision Aid content.*

The working group of the fourth stage consisted of 7 members recruited from the expert panel: 3 cardiologists from different hospitals, 1 decision aid development expert, 1 general practitioner/family medicine physician, 1 internist-geriatrician and 1 dedicated MD PhD-fellow focused on ICD patient care. Of the working group members, 5 (71%) were female. The median age was 55 (IQR 37–58) years and the median clinical experience was 22 (IQR 6–31) years. Working group members together formulated the content of the decision aid, based on current guidelines and literature and tailored it to patient preferences and the Dutch clinical practice based on data gathered in the previous stages.

*Stage 5: Usability testing among patients.*

Four patients participated in the usability testing, of which three were ≥ 70 years. All patients completed the decision aid within half an hour. First impressions of the decision aid were stated to be *inviting*, *clear* and of *additional value*. Three patients appreciated that they could have the opportunity to walk through all the information in a calm home setting. They commented that “it had been impossible to remember all of what the doctor told in the consultation room.” One patient admitted to listen to specific advice from the doctor and read as little as possible about potential complications. Nevertheless, also this patient agreed that the opportunity to be informed on all aspects is beneficial for the general patient group.

## Discussion

This study was designed in order to create a decision aid for ICD patients. Information was gathered systematically on the Dutch clinical practice in ICD patients, patient preferences and insight in ICD therapy, incorporating expert opinions and levels of shared decision making as experienced by patients. Findings were incorporated into the design of a decision aid to support patients and caregivers to make well-informed choices regarding ICD therapy. This evidence-based decision aid was developed for all ICD patients facing the choice of receiving a new ICD or replacing one, according to the Patient Decision Aid Standards (IPDAS) (11) using the RAND-UCLA/multi-stepped Delphi model (12, 13). A previous evidence-based developed ICD decision aid was centered around the health care providers. The main findings in the stages of our study are that: [1] patients in retrospect reported they were not aware of having a choice, [2] levels of shared decision making perceived by our ambulatory ICD population were low and [3] the first patient experience with our decision aid was positive and promising.

### Challenges in patient education and counseling

Patients have a right to be well informed on the various aspects of the proposed intervention, emphasizing the patient’s role in decision making, discussing alternatives and the risks and obtaining the patient’s consent. Moreover, proper counseling of patients is a cornerstone of a prosperous patient-caregiver relationship. The recently updated guidelines of the European Society of Cardiology emphasize the need for patient-centered care and shared decision making (9). Counseling and educating patients on their individual illnesses and therapeutic options can, however, be challenging. This is particularly true for ICD candidates, as not only is it challenging to predict the benefit from an ICD for an individual patient, but an ICD also has its downsides, such as a lower quality of life after receiving ICD therapy (26, 27). This is particularly true in those who have received inappropriate therapy (28). Other important risks include infection, technical failure and receiving shocks in the last moments of life (29–36).

Traditionally, patients are counseled by their caregivers at the outpatient office. These consultations can be supplemented by informative pamphlets filled with information. Or, as a more modern approach, shared decision making with the use of (digital) decision aids can be used.

### Consultations with doctors

It has previously been described that doctors have a decisive role in decision making for patients eligible for an ICD (37). Very strong language emphasizing the benefits of an ICD will lead to patients favoring the device implantation (37, 38). These findings reaffirm the necessity for an unbiased decision aid.

Comprehension by patients of mere percentages has been shown to be overall disappointing (5). ICD patients overestimate the potential benefit from ICD therapy and are deficient in their comprehension of device function (39–43). ICD patients have previously reported to have not fully understood the risks and burden of living with an ICD at the time of consent for an ICD implantation (37, 39). In addition, it has appeared that some patients who had previously declined an ICD implantation for primary prevention, in retrospect had not fully understood the benefits for survival (44).

### Print-based educational material

Patients desire to have access to comprehensive information that can help them in deciding. Providing patients with comprehensive information and considering their preferences, is important for sustainable decision making. Interestingly, traditional print-based educational material for ICD patients has previously been proven to target the highly literate population (45). For this reason, the expert panel decided for the decision aid in this study to be made available online, be interactive and incorporate illustrative educational videos in simple language. To avoid bias toward patients with lower digital literacy, it was, nevertheless, decided that the content can be printed out and handed to patients by healthcare providers in selected cases. In addition, all text was reviewed by professional content writers to be comprehendible for the lower-literate population.

### Shared decision making

The decision making process for the ICD patient is triggered when the risk of sudden cardiac death is discussed (41). However, these ICD patients have reported that the most important factors influencing their final decision were not the odds and numbers, but trust in the advocacy of their treating physician, social influences and their health state (41). Likewise, in stage 2 of the process, patients also reported to have trusted their doctors’ judgment and (strong) recommendations. This illustrates the importance of patient preferences in shared decision making. Moreover, a key factor in shared decision making is helping patients explore preferences and make well thought-out decisions.

In clinical practice, shared decision making is, however, still underutilized. Patients in this study reported relatively low experience of shared decision making. Moreover, most interviewed patients admitted not to have been aware they had a choice. Likewise, patients have previously reported not to recall alternatives for committing to ICD therapy (41). In addition, in a previous study, clinicians have reported that in order to use shared decision making, they needed a hint or trigger from patients, as it was not part of their standard practice (46).

### Shared decision making supplemented by decision aids

Shared decision making can be facilitated by the implementation of decision aids. It has been affirmed that a decision aid results in patients playing a more active role in decision making and accurate risk perception improve patient knowledge and decrease decisional conflict (47). Patients have reported to feel more knowledgeable, better informed, and clearer about their values with the use of a decision aid (47). A pilot study with a decision aid for ICD patients showed promising results, with decrease of decisional conflict in patients using the decision aid (48). Medicare and Medicaid Services in the United States of America, even mandated the use of evidence-based decision aids, supporting shared decision making, in patients that were a candidate for cardiovascular device placements, including ICDs (10). Nevertheless, implementation of decision aids in clinical practice is slow (49). American physicians self-reported to engage in shared decision making when obtaining consent prior to an ICD implantation, however, less than half of these physicians used a decision aid in their clinical practice (50). Lewis at al., developed a user-centered ICD decision aid to be used for patients facing and ICD replacement, involving key-users in the development in order to encourage utilization of the product in the future. In our study, we proactively involved not only cardiologists, but also experts from relevant medical fields and patients. Moreover, the opinion of 233 ambulatory ICD patients has been taken into account when designing this decision aid (Stage 2B).

### Decision making at the time of battery depletion

The expert panel in this study decided to target the ICD decision aid at not only patients eligible for a first ICD, but also patients facing an ICD replacement as ICD therapy is not a lifelong commitment. However, as our patients stated, the latter is not always information that is clear to patients. Moreover, as illustrated in our previous study, ICD replacement was not always presented as a choice by health care providers (14). Likewise, it has been previously shown that more than half of the patients who had already undergone an ICD replacement at the time of battery depletion, had not been aware that they had a choice (51). This illustrates that ICD replacement at the time of battery depletion goes without saying, whereas patients have been reported to consider non-replacement under certain circumstances such as serious comorbidity and advanced age (51).

Time from first ICD implantation to pulse-generation exchange can easily be longer than 5 years (52). Discussions with the healthcare provider and information provided at the commencement of ICD therapy can be forgotten by the patient. Therefore, at the time of pulse-generator exchange for battery depletion, there is a need for renewed discussions with the patient before deciding on definitely continuing ICD therapy. This is in contrast of continuing ICD therapy regardless of the costs (risk of complications) or patient preferences (e.g., no longer wanting to prevent a sudden cardiac death).

Moreover, patient preferences can change with the progression of age and the development of comorbidities. In addition, the odds of complications increase with every pocket revision/redo procedure (52, 53).

An ICD decision aid can also facilitate decision making in these patients, exploring their current individual preferences and weighing them out against the expected benefits and downsides from ICD therapy. Especially a decision aid that can be reviewed at home, will provide an opportunity for family members to be involved in the decision making resulting in decisions supported by patients and their doctors as well as their families.

Previous endeavors resulted in a healthcare-provider-centered ICD decision aid to be implemented in patients facing an ICD replacement, in order to help healthcare providers step away from the automatism of replacing an ICD at battery depletion instead of discussing the options with their patients first (46). It is expected from the decision aid resulting from this study, to encourage not only healthcare providers but also patients into taking a more active role in the decision making process prior to the definitive continuation of ICD therapy.

### Future perspective

The ICD Decision Aid Study is currently being conducted in a multicenter stepped wedge clustered randomized trial at 6 Dutch centers. The study will evaluate the decision aid in a clinical setting and its benefit on shared decision making experienced by both doctors and patients. Shared decision-making levels in our population will be reassessed after implementation, to clarify the benefit of the ICD decision aid.

## Conclusion

This study describes the evidence-based approach to the development of the Dutch ICD Decision Aid. In our population, levels of shared decision-making experience were low. Decision aids have previously proven to improve patients’ decision making and facilitate shared decision making. The ICD Decision Aid was developed for the Dutch ICD patient population according to prevailing decision aid development methods. Results from our multicenter stepped wedge clustered randomized trial will further evaluate the ICD decision aid.

### Limitations

This study has several limitations. Most importantly, recall bias can be present in the patient groups. Patients reporting experience in Stage 2 are prone to recall bias. However, reported outcomes are accurate for evaluating patient experience as this is what patients eventually actually remember. Moreover, patients from the second round of Stage 2 are a good representation of the average ambulant ICD patients, as hospitalized or patients with end-stage disease would not be able to attend.

With regards to Stage 3, there is a selection bias in patients entering the panel and expert group. Participants have, however, been carefully selected on their roles in the clinical field and experience with ICDs. Using 2 rounds in this stage allowed elaborate discussions of their points of view and consensus was reached on all items.

In Stage 5, the usability of the decision aid was tested amongst a small number of selected patients. The evaluation was, however, performed carefully and with much attention. Patients were not pre-selected on their computer skills and included patients of old age. It is expected that this patient group is a good representation of the whole population. In addition, no health care professionals were included in stage 5. The aim was to assess the usability of the digital tool amongst a diverse group of patients. Health care professionals will, however, be included in the upcoming randomized clinical trial, that will evaluate the decision aid within a broad clinical setting.

Finally, the option of a subcutaneous ICD (without transvenous leads) was not included in the current decision aid. The current ICD decision aid is aimed towards helping patients decide whether or not to protect themselves from sudden cardiac death (i.e., choosing for defibrillator therapy). This decision must be made first, before the option of a subcutaneous ICD can be explored. Development of a future decision aid to help patient explore this subsequent option, could be useful in selected cases suited for both a transvenous and a subcutaneous ICD.

## Data availability statement

The raw data supporting the conclusions of this article will be made available by the authors, without undue reservation.

## Ethics statement

The studies involving human participants were reviewed and approved by the Scientific Review Board of the Leiden University Medical Center, Department of Cardiology and Leiden University Medical Center Medical Ethics Committee. All patients and experts involved in panels and interviews for study purposes provided written informed consent to participate in this study.

## Author contributions

DY, LE, MS, RV, HS, and AE contributed to conception and design of the study. DY organized the database and wrote the first draft of the manuscript. DY and LE performed the statistical analysis. All authors contributed to manuscript revision, read, and approved the submitted version.
