# Supplementary material for: The development of a decision aid for shared decision making in the Dutch implantable cardioverter defibrillator patient population: A novel approach to patient education
Source: Front Cardiovasc Med. 2022 Oct 13;9:946404. doi: 10.3389/fcvm.2022.946404 (PMC9606344; doi:10.3389/fcvm.2022.946404)
Supplement: Supplementary file 1 [file Data_Sheet_1.docx]

**Appendix 1**

**Table 1A:** List of all ICD Implanting Dutch Centers included in round 1

| St. Antonius Ziekenhuis, Nieuwegein |
| --- |
| Academic Medical Center, Amsterdam |
| Leiden University Medical Center, Leiden |
| Scheper Hospital, Emmen |
| Medical Spectrum Twente |
| Radboud Medical Center, Nijmegen |
| Spaarne Gasthuis, Haarlem |
| Erasmus MC, Rotterdam |
| Catharina-hospital, Eindhoven |
| Nordwest Hospitalgroup, Alkmaar |
| University Medical Center Groningen, Groningen |
| FlevoHospital, Almere |
| Albert Schweitzer Hospital, Dordrecht |
| Haaglanden Medical Center, The Hague |
| Medical Center Leeuwarden, Leeuwarden |
| Rijnstate Hospital, Arnhem |
| TweeSteden Hospital, Tilburg |
| Canisius-Wilhelmina Hospital, Nijmegen |
| Franciscus Gasthuis & Vlietland, Schiedam |
| Maasstad Hospital, Rotterdam |
| University Medical Center Utrecht, Utrecht |
| HagaZiekenhuis, The Hague |
| Free University Medical Center, Amsterdam |
| Isala Clinics, Zwolle |

**Table 1B:** “White List 2017”: *Dutch hospitals licensed to perform ICD procedures*

| ***ICD-implementing Centers*** |
| --- |
| Academic Medical Center, Amsterdam |
| Albert Schweitzer Hospital, location Dordwijk, Dordrecht |
| Amphia Hospital, location Molengracht, Breda |
| Canisius-Wilhelmina Ziekenhuis, Nijmegen |
| Catharina-Hospital, Eindhoven |
| Erasmus Medical Center, Rotterdam |
| FlevoHospital, Almere |
| Franciscus Gasthuis & Vlietland, Schiedam |
| HagaZiekenhuis, The Hague |
| Isala Clinics, Zwolle |
| Leiden University Medical Center, Leiden |
| Maasstad Hospital, Rotterdam |
| Maastricht University Medical Center, Maastrisch |
| Martini Hospital, Groningen |
| Haaglanden Medical Center, The Hague |
| Nordwest Hospital Group, Alkmaar |
| Medical Center Leeuwarden, Leeuwarden |
| Medical Spectrum Twente, Enschede |
| Onze Lieve Vrouwen Gasthuis, location Oost, Amsterdam |
| Onze Lieve Vrouwen Gasthuis, location West, Amsterdam |
| Radboud University Medical Center, Nijmegen |
| Rijnstate Hospital Arnhem |
| Scheper Hospital, Emmen |
| Spaarne Gasthuis, Haarlem |
| St. Antonius Hospital, Nieuwegein |
| TweeSteden Hospital, Tilburg |
| University Medical Center, Groningen |
| University Medical Center, Utrecht |
| Free University Medical Center, Amsterdam |
