# Supplementary material for: The development of a decision aid for shared decision making in the Dutch implantable cardioverter defibrillator patient population: A novel approach to patient education
Source: Front Cardiovasc Med. 2022 Oct 13;9:946404. doi: 10.3389/fcvm.2022.946404 (PMC9606344; doi:10.3389/fcvm.2022.946404)
Supplement: Supplementary file 2 [file Data_Sheet_2.docx]

**Appendix 2**

**Table 1a:** statements on who should be the target group of the decision aid should be.

| **Statements** |
| --- |
| The decision aid should be given to … |
| *… all patients receiving an ICD.* |
| *... all patients receiving an ICD for the first time.* |
| *… all patients who will undergo a pulse-generator replacement due to battery depletion.* |
| *… only patients receiving an ICD for primary prevention of sudden cardiac death.* |
| *… patients concerning secondary prevention of sudden cardiac death.* |
| *... all patients with many comorbidities.* |
| *... patients of high age.* |
| The decision aid should be handed out by/made available by… |
| *… the cardiologist* |
| *… the general practitioner / family doctor* |
| *… the ICD-nurse* |
| *... the ICD-technician* |
| *... the patient union* |
|  |
| The decision aid should be handed out per postal mail, *before* the consultation with the cardiologist on ICD therapy |
| The decision aid should be handed out *after* consultation with the cardiologist on ICD therapy |
| The decision aid should be given to … |
| *… all patients receiving an ICD.* |
| *... all patients receiving an ICD for the first time.* |
| *… all patients who will undergo a pulse-generator replacement due to battery depletion.* |
| *… only patients receiving an ICD for primary prevention of sudden cardiac death.* |

ICD: implantable cardioverter-defibrillator. Original statements in Dutch.

**Table 1b**: statements on who should be included in the content of the decision aid.

| **Statements** |
| --- |
| - General explanation about what an ICD does should be included in the decision aid |
| - Discussion on therapeutically benefits of an ICD with primary prevention patients should be separate from secondary prevention patients |
| - Explanation of Cardiac Resynchronization Therapy (CRT) should be included to the content |
| - The added value of an ICD with patients at an older age should be discussed nuanced |
| - The added value of an ICD with patients with unclear life expectancy should be discussed |
| - The most common complications of a procedure should be discussed |
| - Complications of a prolonged hospitalization, such as pneumonia and decubitus in case of immobilization, should be discussed |
| - Risk on advisory leads and recall products should be included in the explanation by default |
| - The role of the ICD at the end of life should be discussed with all patients |
| - The possibility to deactivate tachytherapy at the end of life should be discussed with all patients |
| - All patients should know that an ICD should not be a lifelong commitment |
| - All patients should know that an ICD, if not desired, can be turned off |
| - The role of the ICD at the end of life should be discussed with patients of old age |
| - The possibility to deactivate tachytherapy at the end of life should be discussed with patients of old age |
| - Patients of old age should know that an ICD does not have to be a lifelong commitment |
| - Patients of old age should know that an ICD, if not desired, can be turned off |
| - The technical aspects of how an ICD works should be included in the counselling material |
| - The benefits of brachytherapy should be explained |
| - It should be **explained** that ICD therapy protects against sudden cardiac death and not against sudden death in general (because of other causes of death) |
| - It should be **stressed** that ICD therapy protects against sudden cardiac death and not against sudden death in general (because of other causes of death |
| - The psychological impact of brachytherapy (more depressions, traumatic) should be included in the general content |
| - The chance of inappropriate therapy should be included in the content |
| - How you should resuscitate a patient with ICD should be included to the content |
| - Telemonitoring should be explained |
| - The function of various healthcare specialists, cardiologist, EP-cardiologist, ICD-nurse and ICD technician, should be explained in the content |

ICD: implantable cardioverter-defibrillator. EP: electrophysiologist. CRT: cardiac resynchronization therapy. Original statements in Dutch.

**Table 1c**: statements on items to be included in rating scales for patients.

| **Statements** |
| --- |
| In the decision aid, patients should be able to select on a rating scale… |
| …how much they tend to an ICD or not. |
| .. how much they value the advice of their health care provider. |
| … how much they value the opinion of close ones / relatives. |
| … how much anxiety they feel for receiving appropriate therapy. |
| … how much anxiety they feel for receiving inappropriate therapy. |
| … how self-sustainable they will feel when shock therapy is being felt. |
| … how self-sustainable they expect to be in showing up on all follow-up appointments. |
| … how willing they will be to undergo re-inventions for battery replacements. |
| … how affected they will be by the consequences for their driver’s license after implantation. |
| … how affected they will be by the consequences for their driver’s license after receiving shock therapy. |
| … how willing they are to comply with the necessity for at least two-yearly ICD semi-annual |
| … how much anxiety they feel for potential complications |
| … their value for philosophical elements, such as the role of ICD at the end of life. |
| … their value for the psychological aspects of shock therapy, such as the probability of depressions and decrease of quality of life |
| ... their preference for life extension, such as the role of ICD in the mortal process |
| … their value for the cosmetic aspects of an ICD, such as the scar and visibility of the contour of the pulse-generator |
| … their value for psychological aspects of shock therapy, such as the probability of depressions and decrease of quality of life |
| … their preference for life extension above quality of life (for instance: understanding that preventing sudden cardiac death can lead to a long hospitalization with heart failure) |
| …their preference for a non-sudden cardiac death with a potential prolonged death bed. |

ICD: implantable cardioverter-defibrillator. Original statements in Dutch.

**Table 1d**: statements on which patients should be screened on what aspects, by tools integrated into the decision aid.

| **Statement** |
| --- |
| With a tool incorporated into the decision aid, …. |
| **… all** patients should be screened on frailty. |
| **… all** patients should be screened on social-cognitive functions. |
| **… all** patients should be screened for dementia. |
| **... all** patients should be screened on vitality. |
| ... patients older than **65** years should be screened on **frailty**. |
| ... patients older than **65** years should be screened on **social-cognitive functioning**. |
| ... patients older than **65** years should be screened on **dementia**. |
| ... patients older than **65** years should be screened on **vitality**. |
| ... patients older than **70** years should be screened on **frailty**. |
| ... patients older than **70** years should be screened on **social-cognitive functioning**. |
| ... patients older than **70** years should be screened on **dementia**. |
| ... patients older than **70** years should be screened on **vitality**. |
| ... patients older than **75** years should be screened on **frailty**. |
| ... patients older than **75** years should be screened on **social-cognitive functioning**. |
| ... patients older than **75** years should be screened on **dementia**. |
| ... patients older than **75** years should be screened on **vitality**. |
| ... patients older than **80** years should be screened on **frailty**. |
| ... patients older than **80** years should be screened on **social-cognitive functioning**. |
| ... patients older than **80** years should be screened on **dementia**. |
| ... patients older than **80** years should be screened on **vitality**. |

Original statements in Dutch.

**Table 1e:** statements on what kind of medium the decision aid should be available in.

| **Statement** |
| --- |
| The decision aid should be available in a paper version |
| The decision aid should be available as a downloadable app |
| Only web-access to the decision aid will be sufficient |
| An interactive decision aid, including videos of patient experiences is preferable |
| Videos with experiences of other patients does not belong in a decision aid |

Original statements in Dutch.
